# Prevalence of refractive error among Chinese preschool children: The Changsha children eye study

**DOI:** 10.3389/fpubh.2022.1019816

**Published:** 2022-11-22

**Authors:** Yuxia You, Junxia Fu, Ming Xu, Yali Song, Huanfen Zhou, Shihui Wei

**Affiliations:** ^1^Beijing Aier Intech Eye Hospital, Beijing, China; ^2^Aier Eye Hospital Group, Changsha, Hunan, China; ^3^Department of Ophthalmology, The Chinese People's Liberation Army Medical School, The Chinese People's Liberation Army General Hospital, Beijing, China; ^4^Department of Ophthalmology, Xinhua Hospital Affiliated to Shanghai Jiao Tong University School of Medicine, Shanghai, China

**Keywords:** refractive error, preschool children, prevalence, myopia, myopia epidemiology

## Abstract

**Purpose:**

We aimed to investigate the refractive status and prevalence of refractive error, as well as its characteristics in Chinese preschool children aged 1–6 years old.

**Methods:**

A population-based cross-sectional study—Changsha Children Eye Study (CCES) was conducted. The prevalence of refractive errors among children aged 1–6 years old from 18 community health service centers was surveyed. A handheld child vision screener, Suowei, was used for examination.

**Results:**

A total of 43,105 preschool children were included. The mean spherical equivalent (SE) was 0.42 ± 1.05 D for the right eyes. The mean astigmatism (diopter of cylinder, DC) was −0.83 ± 1.02 D for the right eyes. The magnitude of refractive error was lower in older children, indicating the ongoing of the emmetropization during the 1–6-year-old children. The prevalence of myopia (SE ≤ −1.00 D), hyperopia (SE ≥ +2.00 D) and astigmatism (DC ≥1.50 D) was 2.94, 13.8 and 17.6%, respectively. The prevalence of myopia decreased with the increase of age between the six age groups (*P* < 0.001). The prevalence of hyperopia was lower in 5–6 years old, whereas, the prevalence of myopia was slightly higher at this period of time. With-the-rule (WTR) astigmatism (+ cylinder axis 90° ± 15°) was the most prevalent type of astigmatism than against-the-rule (ATR) astigmatism (+ cylinder axis 180° ± 15°) and oblique (OBL) astigmatism (*X*^2^ = 209.5, *P* < 0.001). The binary logistic regression model showed that older age and suffering astigmatism were independently associated with the development of myopia. In addition, there was no significant gender difference in the prevalence of myopia, emmetropia, and hyperopia.

**Conclusions:**

Our population-based cross-sectional study investigated the prevalence of myopia, hyperopia, and astigmatism in preschool children aged 1–6 years old. The distribution of the refractive error was disperse in the younger group and gradually turned more centralized in older group. Similar to hyperopia, with age increased, the prevalence of myopia was lower in preschool children younger than 5 years old and then slightly increased at 5–6 years, which may indicate an early sign of myopia in school-age children. Therefore, we emphasize that more attention should be given to the children at this age.

## Introduction

Myopia has emerged as a significant health issue (1). The prevalence of myopia has increased rapidly over the past few decades worldwide (2, 3). It is reported that ~60% of 12-year-old primary school students, 80% of 16-year-old high school students, and more than 90% of university students have myopia in China (4–6). Myopia mainly occurs in school-age children (7), and for children under 6 years old, the prevalence of myopia is relatively lower (8–11).

Many studies reported the refractive status of school-aged children in the world (12–14), however, only a few studies focused on the refractive status of preschool children, especially in China. The uncorrected refractive error among preschool children could raise the higher risk of amblyopia and strabismus. Furthermore, preschool children with amblyopia and or strabismus have a high risk of developing high myopia, leading to secondary irreversible blinding complications (15–18). It might become a huge economic burden for both family and society. As the visual system is not fully developed, the preschool myopia might share different risk factors (19–21) with school-age myopia (22–24). To better understand the refractive status among preschool children, we performed a cross-sectional study. We aimed to investigate the refractive status and prevalence of refractive error, as well as its characteristics in Chinese preschool children aged from 1 to 6 years old.

## Methods

The Changsha Children Eye Study (CCES) is a population-based study to estimate the prevalence and risk factors for refractive error and ocular diseases (25). The study was approved by the ethics committee of Beijing Aier Intech Eye Hospital. The data were obtained through the Mulin telemedicine platform (Hunan Super Vision Technology Co., Ltd.).

This study was performed from April 2016 to July 2019 among children aged 1–6 years from 18 community health service centers in Changsha, China. The consent to participate in the study was obtained from children's parents or their legal guardians. Children with systemic diseases such as congenital heart diseases and ocular trauma or eye diseases such as glaucoma, cataracts, and strabismus were excluded.

The examination workflow of CCES was as follows: a handheld child vision screener Suowei (Tianjin Suowei Electronic Technology Co., Ltd.) was used to screen children's binocular refractive condition (26). The examination was routinely conducted by a general practitioner in a dark room. Before the study, all the general practitioners were trained by ophthalmologists in terms of conducting standard eye examinations and using the handheld child vision screener. The binocular sphere, astigmatism, pupil size, pupillary distance, and fixation direction were obtained, recorded, and uploaded to the Mulin telemedicine platform.

The children were categorized into 6 groups by their age at the first examination: 1-year-old group (0 < age ≤ 12 months), 2-year-old group (1 < age ≤ 2 years), 3-year-old group (2 < age ≤ 3 years), 4-year-old group (3 < age ≤ 4 years), 5-year-old group (4 < age ≤ 5 years), 6-year-old group (5 < age ≤ 6 years).

The refractive error was evaluated using the spherical equivalent (SE), calculated by adding the sum of the sphere power with half of the cylinder power. In previous studies, SE ≤ −0.50 D or SE ≤ −1.0 D after cycloplegia is defined as myopia, and SE ≥ +2.00 D is defined as hyperopia. In our study, considering that none of the participants underwent cycloplegia, we used three thresholds (SE ≤ −50 D, SE ≤ −1.00 D and SE ≤ −2.00 D) to calculate the prevalence of myopia to reduce the false positive rate. The data of right eyes were taken to calculate the prevalence of myopia. The definition of hyperopia was SE ≥+2.00 D. The definition of astigmatism was the cylinder power ≥1.50 D and astigmatism was classified into three categories: with-the-rule (WTR) (+ cylinder axis 90° ± 15°), against-the-rule (ATR) (+ cylinder axis 180° ± 15°), and all the other orientations were considered oblique (OBL) (9).

The statistical analyses were performed with an open-source R-program (version 3.6.2) and SPSS software (IBM-SPSS, V 16.0). The mean value and standard deviation were used for the statistical description of continuous variables, and frequency and percentage were used for the statistical description of categorical variables. The univariate analysis was performed by the chi-square test or chi-square trend test. The multivariable analysis was performed by the bivariate logistics analysis. The odds ratio (OR) value of each variable with the corresponding 95% confidence interval (95% CI) was calculated. *P* < 0.05 was considered statistically significant.

## Results

### Basic characteristics

**Figure 1:**
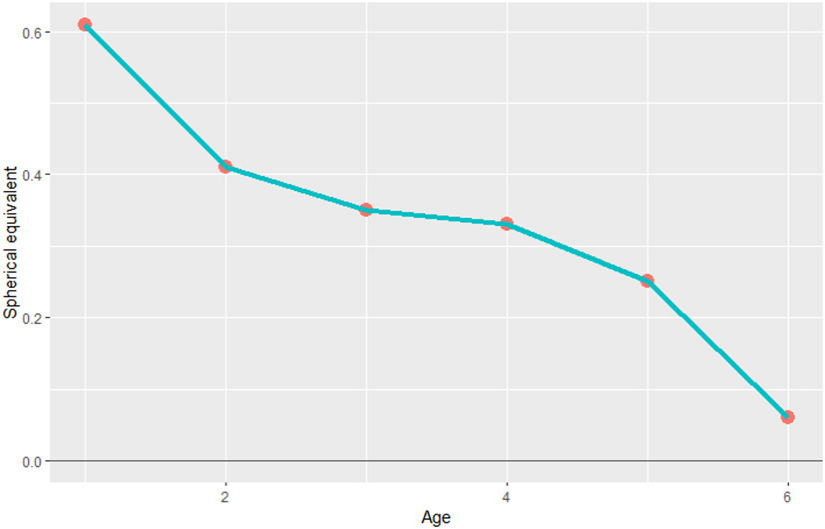
The mean SE among the different age groups. SE, spherical equivalent.

A total of 43,105 children were enrolled in our study (Table 1). 35.8% of the children were in the 1-year-old group, 16.51% were in the 2-year-old group, 14.09% were in the 3-year-old group, 15.30% were in the 4-year-old group, 11.98% were in the 5-year-old group, and 6.29% were in the 6-year-old group. The number of boys and girls was roughly balanced among all age groups. The mean SE of preschool children was 0.42 ± 1.05 D for right eyes. The mean astigmatism (DC) was −0.83 ± 1.02 D for right eyes (Table 1). As shown in Figure 1, the mean SE is lower in older groups. This tendency indicated that the emmetropization is ongoing among children aged 1–6 years. Mean astigmatism is lower between the ages of 1 and 2 and stayed relatively stable between the age of 2 and 4. The mean value of astigmatism was higher in the group of 4-year-old and 6-year-old, reaching ~-0.65 D at six years old (Table 2).

**Table 1:** Refractive error of the 1-6 years old children in the Changsha Children Eye Study.

**Age group (-year-old)**	**Number**	**Percentage (%)**	**SE (mean ±SD)**	**DC** **(mean ±SD)**
1	15,451	35.85	0.61 ± 1.20	−1.02 ± 0.78
2	7,115	16.51	0.41 ± 1.03	−0.75 ± 0.67
3	6,073	14.09	0.35 ± 0.93	−0.77 ± 2.02
4	6,593	15.30	0.33 ± 0.95	−0.72 ± 0.68
5	5,163	11.98	0.25 ± 0.85	−0.67 ± 0.65
6	2,710	6.29	0.06 ± 0.68	−0.64 ± 0.64
Total	43,105	100	0.42 ± 1.05	−0.83 ± 1.02

n, number; SE, spherical equivalent; DC, astigmatism; SD, standard deviation.

**Table 2:** Prevalence of different astigmatism types of the 1-6 years old children in the Changsha Children Eye Study.

**Age(-year-old)**	**WTR (n, %)**			**ATR (n, %)**			**Oblique (n, %)**		
	**Boy**	**Girl**	**All**	**Boy**	**Girl**	**All**	**Boy**	**Girl**	**All**
1	1,403 (17.3)	1,435 (19.5)	2,838 (18.4)	116 (1.4)	91 (1.2)	207 (1.3)	604 (7.5)	522 (7.1)	1,126 (7.3)
2	289 (7.8)	297 (8.7)	586 (8.2)	47 (1.3)	47 (1.4)	94 (1.3)	154 (4.2)	127 (3.7)	281 (4.0)
3	573 (9.4)	304 (9.6)	269 (9.3)	21 (0.7)	13 (0.4)	34 (0.6)	93 (2.9)	85 (2.9)	178 (2.9)
4	325 (9.1)	287 (9.4)	612 (9.3)	10 (0.3)	12 (0.4)	22 (0.3)	90 (2.5)	80 (2.6)	170 (2.6)
5	255 (9.3)	230 (9.6)	485 (9.4)	9 (0.3)	6 (0.2)	15 (0.3)	51 (1.9)	43 (1.8)	94 (1.8)
6	117 (7.7)	96 (8.1)	213 (7.9)	3 (0.2)	3 (0.3)	6 (0.2)	20 (1.3)	25 (2.1)	45 (1.7)

n, numbers, WTR, with-the-rule; ATR, against-the-rule.

According to the outcome of the chi-square test, the prevalence of WTR is significantly greater than that of ATR and Oblique (X^2^ = 209.5, P < 0.001). The boy had a higher prevalence of astigmatism than the girl (X^2^ = 209.5, P < 0.001). Moreover, with the increase of age, the prevalence of astigmatism showed a descending tendency which has statistical significance (P < 0.001).

### Prevalence of myopia

**Figure 2:**
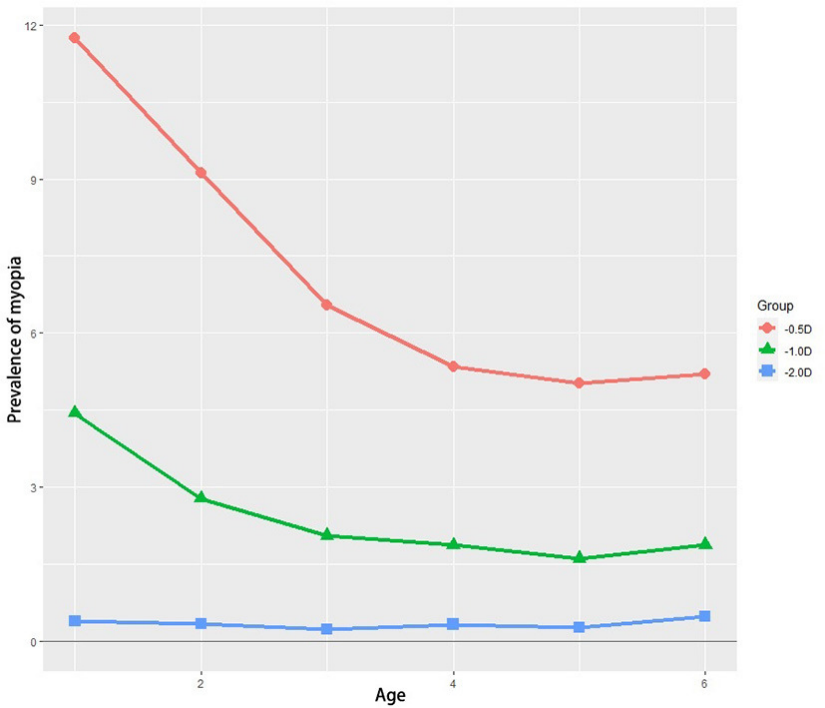
The prevalence of myopia that is defined by three thresholds among the six different age groups. D, diopter.

The prevalence of myopia was 8.39%, 2.94%, 0.34% with the definition of SE ≤ −0.50 D, SE ≤ −1.00 D, and SE ≤ −2.00 D, respectively (Table 3). In the group of SE ≤ −0.50 D and SE ≤ −1.00 D, the prevalence of myopia decreased as age increased (Figure 2). Whereas in the group of SE ≤ −2.00 D, the prevalence of myopia was relatively lower and stable among all age groups. Moreover, no significant difference was found in the prevalence of myopia between genders in all age groups (*p* ≥ 0.05, Table 4). The binary logistic regression model showed that older age and suffering from astigmatism were independently associated with the development of myopia (Table 5).

**Table 3:** The prevalence of myopia with different thresholds and astigmatism in 1–6 years old children.

**Age**	***n***	**Myopia (** ***n*** **, %)**			**Astigmatism (*n*, %)**
		**≤ -0.50 D**	**≤ -1.00 D**	**≤ -2.00 D**	**≥1.50 D**
1	15,451	1,815 (11.75%)	686 (4.44%)	60 (0.39%)	4,171 (27.00%)
2	7,115	649 (9.12%)	198 (2.78%)	24 (0.34%)	961 (13.51%)
3	6,073	398 (6.55%)	125 (2.06%)	14 (0.23%)	784 (12.91%)
4	6,593	353 (5.35%)	123 (1.87%)	22 (0.33%)	804 (12.19%)
5	5,163	259 (5.02%)	83 (1.61%)	14 (0.27%)	594 (11.50%)
6	2,710	141 (5.20%)	51 (1.88%)	13 (0.48%)	264 (9.74%)
Total	43,105	3,615(8.39%)	1,266 (2.94%)	147(0.34%)	7,578 (17.58%)

n, number; D, diopter.

**Table 4:** The proportion of myopia, emmetropia, and astigmatism with different thresholds in 1–6 years old children.

**Age(-year-old)**	***n***	**Myopia (** ≤ **-1.0 D**, ***n*****, %)**			**Emmetropia (** ≤ **2.0 D and** ≥**-1.0 D**, ***n*****, %)**			**Hyperopia(**≥**2.0 D**, ***n*****, %)**		
		**Boy**	**girl**	**All**	**Boy**	**girl**	**All**	**Boy**	**girl**	**All**
1	15,451	368(2.4%)	318(2.1%)	686 (4.4%)	6,182(40.0%)	5,458(35.3%)	11,640 (75.4%)	1,650(10.7%)	1,474(9.5%)	3,124 (20.2%)
2	7,115	100(1.4%)	98(1.4%)	198 (2.8%)	3,223(45.3%)	2,781(39.1%)	6,004(84.4%)	483(6.8%)	428(6.0%)	911 (12.8%)
3	6,073	66(1.1%)	59(1.0%)	125 (2.1%)	2,779(45.8%)	2,533(41.7%)	5,312(87.5%)	325(5.4%)	309(5.1%)	634 (10.4%)
4	6,593	71(1.1%)	52(0.8%)	123 (1.9%)	3,095(46.9%)	2,617(39.7%)	5,712(86.6%)	386(5.9%)	362(5.5%)	748 (11.4%)
5	5,163	52(1.0%)	31(0.6%)	83 (1.6%)	2,457(47.6%)	2,161(41.9%)	4,618(89.4%)	245(4.7%)	215(4.2%)	460 (8.9%)
6	2,710	30(1.1%)	21(0.8%)	51 (1.9%)	1,457(53.8%)	1,148(42.4%)	2,605(96.1%)	34(1.3%)	20(0.7%)	54 (2.0%)
Total	43,105	687(1.6%)	579(1.3%)	1,266 (2.9%)	19,193(44.5%)	16,707(38.8%)	35,900(83.3%)	3,123(7.2%)	2,808(6.5%)	5,931 (13.8%)

According to the outcome of the Chi-square trend test, the prevalence of hyperopia showed a descending tendency with the increase of age, which has statistical significance (P < 0.001). Instead, children with emmetropia have tapered off. Moreover, the prevalence of myopia decreases with the increase of age, and the tendency has statistical significance (P < 0.001). In addition, there was no significant gender difference in the prevalence of myopia, emmetropia, and hyperopia.

**Table 5:** The prevalence of myopia and astigmatism with different thresholds in 1–6 years old children.

**Characteristic**	**Subgroup**	**OR (95%CI)**	***P*-value**
Gender	Boy	1.00	
	Girl	0.92 (0.82,1.026)	0.131
Age	1-years old	1.00	<0.001
	2-years old	0.82 (0.70,0.97)	0.021
	3-years old	0.61 (0.50,0.74)	<0.001
	4-years old	0.56 (0.46,0.68)	<0.001
	5-years old	0.49 (0.39,0.62)	<0.001
	6-years old	0.61 (0.45,0.81)	0.001
Astigmatism	No	1.00	
	Yes	5.03 (4.48,5.65)	<0.001

OR, Odds ratio; CI, Confidence interval.

The binary logistic regression model showed that older age and suffering from astigmatism were independently associated with the development of myopia. The results are shown in Table 4. As the Collinearity diagnostics showed, the VIF (variance inflation factor) was 2.63 < 10. Therefore, there was no multicollinearity among these factors.

### Prevalence of hyperopia

**Figure 3:**
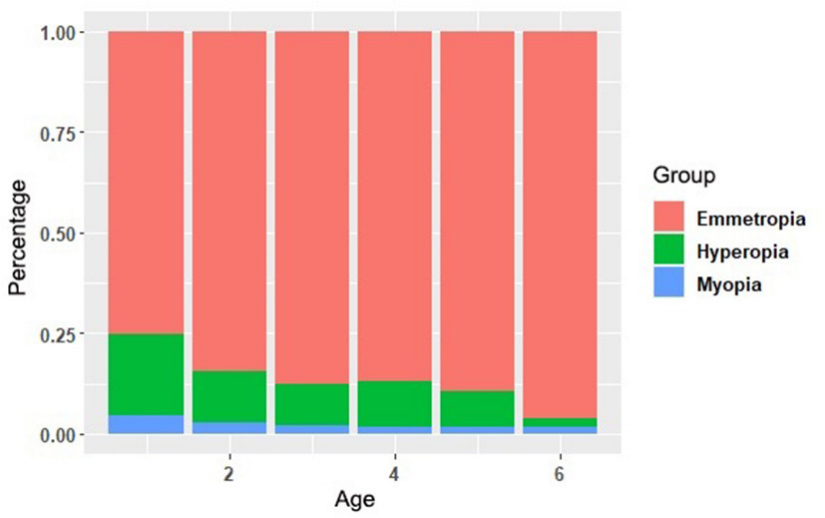
The proportion of emmetropia, hyperopia, and myopia.

As shown in Table 4, the prevalence of hyperopia (SE ≥ 2.00 D) was 13.8%. According to the outcome of the Chi-square trend test, the prevalence of hyperopia showed a descending tendency with the increase of age, which has statistical significance (*P* < 0.001). Instead, children with emmetropia have tapered, and there was no significant difference in the prevalence of hyperopia between different genders. The proportion of myopia, emmetropia and hyperopia was shown in Figure 3. In addition, there was no significant gender difference in the prevalence of myopia, emmetropia and hyperopia (Table 4).

### Prevalence of astigmatism

**Figure 4:**
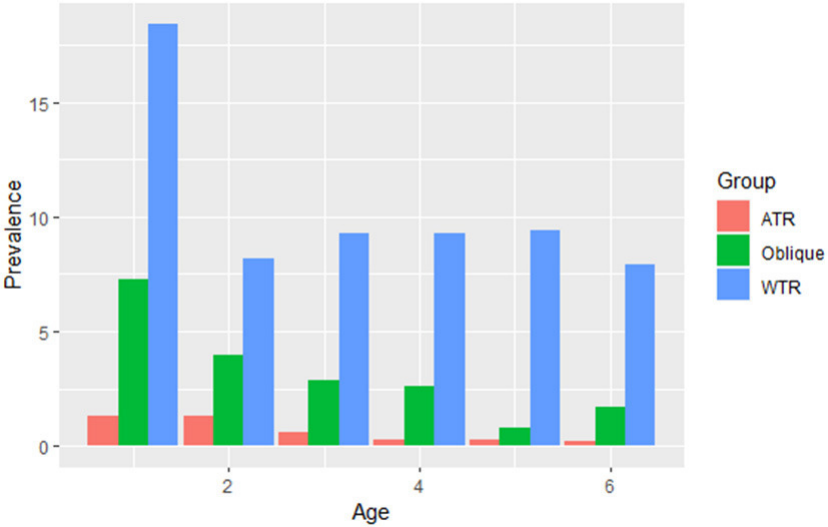
The prevalence of different types of astigmatism among the different age groups. WTR: with the rule (+ cylinder axis 90° ± 15°); ATR: against the rule (+ cylinder axis 180° ± 15°); all other orientations were considered oblique (OBL).

The prevalence of astigmatism (DC ≥ 1.50 D) was 17.6%. Similarly, with the increase in age, the prevalence of astigmatism showed a descending tendency among all age groups, which has statistical significance (*P* < 0.001, Figure 4). With-the-rule (WTR) astigmatism (+ cylinder axis 90° ± 15°) was the most common type of astigmatism than against-the-rule (ATR) (+ cylinder axis 180° ± 15°) and oblique (OBL) (*X*^2^ = 209.5, *P* < 0.001). According to the outcome of the chi-square test, the prevalence of WTR is significantly greater than that of ATR and Oblique (*X*^2^ = 209.5, *P* < 0.001). The boy had a higher prevalence of astigmatism than the girl (*X*^2^ = 209.5, *P* < 0.001, Table 2).

## Discussion

Our study was based on the vision screening of 43,105 preschool children from 18 community health service centers in the urban area of Changsha. As the capital city of Hunan Province, Changsha is an economically important key city in China. The GDP per capita of Changsha in 2019 was 20,000 U.S. dollars, stably in the top-ranking of Hunan province, as well as in the leading position than majority of the cities in China.

In this study, we found that the prevalence of hyperopia and myopia was higher in younger groups of preschool children, and the refractive distribution of preschool children was gradually getting to the average level as the children grew up.

It is well accepted that most infants are hyperopes and the degree of hyperopia gradually declines with age until the completion of emmetropization, which is the similar process that happens in the myopic infants due to the rounder lenses (27, 28). Similarly, our study showed that the mean SE of children aged 1–6 years in CCES was positive and gradually decreased from 0.61 D at 1-year-old to 0.06 D at 6-year-old. The peak of the magnitude of refractive error stayed in positive. The distribution of the magnitude of the refractive error was more disperse in younger groups and more centralized in older groups. The values of refractive error gradually centralize to zero, which was most evident in the first year after birth, indicating significant emmetropization.

The average prevalence of myopia in preschool children in CCES was 8.39%, 2.94%, and 0.34%, with the threshold of SE ≤ −0.50 D, SE ≤ −1.00 D, and SE ≤ −2.00 D, respectively. If we defined SE ≤ −1.00 D as the criteria of myopia to reduce the false-positive rate, the average prevalence of myopia in preschool children in our study was 2.94%. Myopia prevalence in other previous studies around the world was variable (Table 6). Wen G et al. reported that the myopia prevalence was only 1.2% in non-Hispanic whites (aged 6–72 months) in the MEPEDS study (29). However, the STARS study showed that the average prevalence of myopia in Chinese children from 6 to 72 months in Singapore was as high as 11.0% (11), while the prevalence of myopia of 6-year-old children in Taiwan was 6% (30). When we compared our results with the studies in other regions of China, we found that, in this study, the average prevalence of myopia of children aged 1–6 years was 2.94% and was 1.61% for 5-year-old children, which was slightly lower than the children with corresponding age in Hong Kong (31), Taiwan (32) and Shanghai (33). Neverthelss, it is relatively higher than that in Chongqing (34). This might attribute to the different economic levels of these regions. In our study, only the children in urban areas were included, while the Chongqing study focused more on rural areas, and the Shandong study included children both from urban and rural areas.

**Table 6:** Studies about prevalence of myopia among preschool children (%).

**Study**	**Ethnic**	**Numbers**	**Cycloplegia**	**Definition of Myopia**	**Prevalence (%)**					
				**(SE)**	**1Y**	**2Y**	**3Y**	**4Y**	**5Y**	**6Y**
MEPEDS	African-American	2994	1% cyclopentolate or 0.5% cyclopentolate was used in children ≤ 12-month-old	≤ -1.00 D	13.7	9.1	6.4	5.5	4.2	4.1
	Hispanic	3030			6.4	7.2	4.0	1.5	1.8	2.4
	Non-Hispanic white	1501			1.20 (mean)					
	Asian-American	1507			3.98 (mean)					
BPEDS	African-American	1268	1% cyclopentolate or 0.5% cyclopentolate was used in children ≤ 12-month-old	≤ -1.00 D	9.6	7.5	10.5	5.9	6.2	6.6
	Non-Hispanic white	1303			0.0	2.3	1.1	0.0	1.5	1.2
STARS	Singaporean Chinese	2639	Yes, detail is not described	≤ -0.50 D	15.8	14.9	20.2	8.6	7.6	6.4
Japan	Japanese	17320	No	≤ -0.50 D	-	-	-	-	-	3.0
Hong Kong	Chinese	108	1% cyclopentolate	≤ -0.50 D	-	-	-	-	4.6	-
Taiwan	Chinese	618	1% tropicamide	≤ -0.50 D	-	-	3.0	4.2	4.7	12.2
Shanghai	Chinese	5532	0.5% proparacaine/ 1% cyclopentolate	≤ -0.50 D	-	-	1.8	2.3	3.5	5.2
Shandong	Chinese	6026	1% cyclopentolate	≤ -0.50 D	-	-	-	1.7	0.8	4.1
Guangzhou	Chinese	495	1% cyclopentolate	≤ -0.50 D	-	-	-	-	3.3	-
Guangzhou	Chinese	2480	1% cyclopentolate	≤ -0.50 D	-	-	2.1	0.9	0.2	1.6
Chongqing	Chinese	3070	1% cyclopentolate	≤ -0.50 D	-	-	-	-	-	0.4
CCES *	Chinese	43,105	No	≤ -1.00 D	4.4	2.8	2.1	1.88	1.6	1.9

SE, spherical equivalent; Y, -year-old; MEPEDS, the Multi-Ethnic Pediatric Eye Disease Study; BPEDS, the Baltimore Pediatric Eye Disease Study; STARS, The Strabismus, Amblyopia and Refractive Error in Singaporean Children; NA, not available; CCES, The Changsha Children Eye Study; Asterisk indicates our study.

Notably, we found that the prevalence of myopia in preschool children decreased with age, particularly evident from birth to 2 years, then it stayed relatively stable between 2 and 5 years, and followed by a slight increase at 5- to 6-year-old. This trend is similar to the MEPEDS study among African-American and Hispanic-American children (10) and the other two studies (27, 31). Unlike the prevalence of myopia among preschool children, the prevalence of myopia in school-age children increases steadily (5, 35). What is more, emmetropization in infancy is widely acknowledged to decline from farsightedness. However, our study indicated that the emmetropia process could be from nearsightedness to emmetropization too. The nearsightedness in infants and young children might be due to the convex lens shape in the early stage. Although the ocular axis is getting longer with the increase of age, the lens is becoming thinner. Therefore, the refractive status tends to shift from myopia to emmetropia. In addition, it is reported that emmetropization is most obvious within the age of two, and it usually completes before 4 years old (36, 37). Therefore, the slight increase in the prevalence of myopia between 5 and 6-year-olds might be an early sign of myopia in school-age children.

In our study, the prevalence of hyperopia in preschool children was 13.76% and it decreased with age. It is lower than that of the African descent children (20.8%) and Hispanic (26.9%) in the United States (10), but higher than the average prevalence of hyperopia in Chinese children in Singapore MEPEDS study (7.5%), and the 5-year-old children in Taiwan study (4.7%) (Table 6). Moreover, there were two periods of time when the hyperopia were significantly lower than other age group: the one is from 1-year-old (20.22%) to 2-year-old (12.80%), which might be due to emmetropization. The other one is 5-year-old (8.91%) to 6-year-old (1.99%). The reason might be that children at this age are usually ready for school, and more near-distant work is required in their daily life. Besides the physiological emmetropization, we strongly insist that more attention should be paid, both from parents and clinical doctors to myopia monitoring with children aged 5–6 years old.

The prevalence of astigmatism has been reported to vary from 3.8 to 50% in different studies (11). However, in our study, the prevalence of astigmatism among preschool children was 17.6%, and it was lower in older age group. It distinctly dropped from 27.0% at 1 year old to 13.51% at 2 years old, followed by a slow decrease to 9.74% at 6 years old. The prevalence of astigmatism in our study is much lower than that of the Shandong study (36.3%) (5), but higher than that of the Singapore STARS study (8.6%) (11). It is close to another study (19.2%, ages 7–9) in Singapore (17) and the MEPEDS and BPEDS studies (11.4%) for white children. WTR astigmatism was by far the most common form in all age groups, similar to the population-based MEPEDS, BPEDS and STARS studies.

In this study, we included 43,105 preschool children, and 28,639 of them were under three years. To our knowledge, it was an unprecedently enormous sample of the Chinese population with this period of age. However, there were several limitations in the study. Firstly, a cross-sectional study, which, to some extent, indicated the age-related trends, such as emmetropization, was not as reliable as cohort studies. Secondly, due to the difficulty in the practice of cycloplegia, we defined SE ≤ −1.00 D as myopia, which would reduce the false myopia rate, but bias still exists. Thirdly, the younger children group, especially the 1-year-old group, has poor cooperation, which could lead to deviations. Lastly, though many studies have confirmed that automated refraction and retinal retinoscopy are highly correlated, they are different and unavoidable deviations might exist when we use handheld child vision screeners for examination.

Overall, our population-based cross-sectional study investigated the prevalence of myopia, hyperopia, and astigmatism in preschool children aged 1–6 years. We observed that the distribution of the refractive error was disperse in the younger group and gradually turned more centralized in older group. Similar to hyperopia, with age increased, the prevalence of myopia decreased in preschool children younger than 5 years old and then slightly increased at 5–6 years, which may indicate an early sign of myopia in school-age children. According to our study, two periods of time require more attention. One is from 1 to 2 years old, which is a rather physiological change stage when the prevalence of myopia, hyperopia, and astigmatism decreases significantly, indicating that the refractive error within 1 year is likely to decrease at 2 years old. The other one is 5–6 years old, in which the prevalence of hyperopia was lower and the prevalence of myopia was higher, that alarms us to monitor and prevent potential early myopia at this time.

## Data availability statement

The raw data supporting the conclusions of this article will be made available by the authors, without undue reservation.

## Ethics statement

The studies involving human participants were reviewed and approved by the Ethics Committee of Beijing Aier Intech Eye Hospital. Written informed consent to participate in this study was provided by the participants' legal guardian/next of kin.

## Author contributions

YY: data curation, investigation, methodology, writing—original draft, and writing—review and editing. JF: data curation and writing—original draft. MX: data curation, methodology, and supervision. YS: data curation, investigation, and methodology. HZ: methodology, supervision, and writing—review and editing. SW: conceptualization, project administration, supervision, and writing—review and editing. All authors contributed to the article and approved the submitted version.

## Conflict of interest

The authors declare that the research was conducted in the absence of any commercial or financial relationships that could be construed as a potential conflict of interest.

## Publisher's note

All claims expressed in this article are solely those of the authors and do not necessarily represent those of their affiliated organizations, or those of the publisher, the editors and the reviewers. Any product that may be evaluated in this article, or claim that may be made by its manufacturer, is not guaranteed or endorsed by the publisher.
